# Knockdown of lncRNA H19 suppresses endometriosis *in vivo*

**DOI:** 10.1590/1414-431X202010117

**Published:** 2021-02-26

**Authors:** Songping Liu, Weijuan Xin, Qi Lu, Xiaoyan Tang, Fengqin Wang, Wei Shao, Yajiao Zhang, Junjun Qiu, Keqin Hua

**Affiliations:** 1 Department of Obstetrics and Gynecology, Jinshan Hospital of Fudan University, Shanghai, China; 2 Department of Gynecology, Obstetrics and Gynecology Hospital of Fudan University, Shanghai, China; 3 Department of Obstetrics and Gynecology, Zhenjiang Maternal and Child Health Hospital, Zhenjiang, Jiangsu, China

**Keywords:** Endometriosis, lncRNA H19, Gene knockdown, Animal experiments

## Abstract

The long noncoding RNA (lncRNA) H19 is involved in the pathogenesis of endometriosis by modulating the proliferation and invasion of ectopic endometrial cells *in vitro*, but related *in vivo* studies are rare. This study aimed to investigate the role of lncRNA H19 in a nude mouse model of endometriosis. Ectopic endometrial stromal cells (ecESCs) were isolated from ectopic endometrium of patients with endometriosis and infected with lentiviruses expressing short hairpin RNA (shRNA) negative control (LV-NC-shRNA) or lncRNA-H19 shRNA (LV-H19-shRNA). The ecESCs infected with LV-NC-shRNA and LV-H19-shRNA were subcutaneously implanted into forty 6- to 8-week-old female nude mice. The size and weight of the endometriotic implants were measured at 1, 2, 3, and 4 weeks after implantation and compared, and lncRNA H19 levels in endometriotic implants were evaluated using real-time polymerase chain reaction (RT-PCR). All nude mice survived the experimental period, and no significant differences in body weight were observed between the experimental group and the control group. All nude mice developed histologically confirmed subcutaneous endometriotic lesions with glandular structures and stroma after 1 week of implantation. The subcutaneous lesions in the LV-NC-shRNA group after 1, 2, 3, and 4 weeks of implantation were larger than those in the LV-H19-shRNA group, and lncRNA H19 levels in subcutaneous lesions in the LV-NC-shRNA group were significantly higher than those in the LV-H19-shRNA group. Knockdown of lncRNA H19 suppresses endometriosis *in vivo*. Further study is required to explore the underlying mechanism in the future.

## Introduction

Endometriosis is a prevalent gynecological disorder that affects 10-15% of women of reproductive age (1,2). It is defined as the presence and growth of endometrial tissue outside of the uterine cavity and often causes chronic pelvic pain, infertility, menstrual disorders, and pelvic masses (3–5). Endometriosis is a benign disorder but has a high recurrence rate after treatment (6) and may develop into ovarian endometrioid and clear cell cancer (7,8). Although a number of studies have been carried out to understand the etiology of endometriosis, its detailed pathogenesis remains unclear (9–13).

Long noncoding RNAs (lncRNAs) are a group of noncoding single-stranded RNAs with more than 200 nucleotides that participate in biological processes, including cell proliferation, differentiation, chromosome remodeling, epigenetic regulation, transcription, and posttranscriptional modification (14,15). In recent years, many studies have focused on defining the regulatory functions of lncRNAs and found that lncRNAs play important roles in the pathogenesis of many diseases (16–19). However, there are few reports on the involvement of lncRNAs in endometriosis.

In our prior study, we analyzed the endometrial transcriptome in patients with endometriosis using RNA sequencing technology and found that lncRNA H19 had the highest upregulation in both ectopic and eutopic endometrium (20). Further study showed that downregulation of lncRNA H19 could inhibit ectopic endometrial cell proliferation and invasion by modulating miR-124-3p and ITGB3 *in vitro* (21). These studies provide insight into the role of lncRNA H19 in the pathogenesis of endometriosis; however, the function of lncRNA-H19 in endometriosis was not further verified *in vivo*.

In this study, lentiviruses carrying short hairpin RNA (shRNA) negative control (LV-NC-shRNA) or lncRNA H19 shRNA (LV-H19-shRNA) were generated and used to knock down lncRNA H19 levels in ectopic endometrial stromal cells (ecESCs). EcESCs infected with LV-NC-shRNA and LV-H19-shRNA were subcutaneously implanted into female nude mice, after which subcutaneous lesions were observed, and lncRNA H19 levels in lesions were determined. Our study is the first to demonstrate the function of lncRNA H19 in endometriosis *in vivo*.

## Material and Methods

### Patients and samples

Ectopic endometrium samples were collected in September 2018 from 4 patients who had histological evidence of endometriosis. Four patients aged 25-34 years had ovarian cysts that persisted for more than 3 months and were larger than 4 cm in diameter. Surgery was used as the initial treatment, and none of the patients had received hormonal therapy for at least 6 months prior to surgery. All patients had regular menstrual cycles and underwent laparoscopic cystectomy in the late proliferative phase. The clinical characteristics of the patients are shown in Table 1. Institutional review board approval was obtained from Zhenjiang Maternal and Child Health Hospital (No. 20161204), and all patients provided informed consent for the investigation and experiments. Animal studies were reported in compliance with the ARRIVE guidelines (22), and all animal care procedures were followed in accordance with the guidelines set by the European Communities Council Directive (86/609/ EEC).

**Table 1:** Clinical characteristics of the patients with endometriosis.

Characteristics	Patient 1	Patient 2	Patient 3	Patient 4
Age, years	25	29	26	34
Gravidity, n	1	0	1	2
Parity, n	0	0	1	1
BMI, kg/m^2^	22.6	21.4	22.8	24.7
Dysmenorrheal	Present	Absent	Absent	Present
Dyspareunia	Absent	Present	Absent	Absent
Infertility	Present	Present	Absent	Absent
CA125 level (U/mL)	86.4	137.1	34.2	65.9
rAFS stage	III	IV	III	III
DIE status	Absent	Absent	Absent	Absent

BMI: body mass index; rAFS: revised American Fertility Society; DIE: deep infiltrating endometriosis.

### Cell isolation, culture, and identification

Fresh endometriotic tissues were collected during the laparoscopies and washed twice with sterile phosphate-buffered saline (PBS) to remove blood and clots. The cyst walls were minced into 3-5 mm pieces and incubated with 0.25% collagenase type IV (Sigma-Aldrich, USA) in a water bath at 37°C for 90 min. EcESCs were obtained by filtering with a 40-μm monofilament nylon mesh. The digestion and filtration steps were repeated 3 times, and the collected ecESC suspension was centrifuged at 300 *g* for 10 min at 25^o^C. EcESCs were grown in high glucose Dulbecco's modified Eagle medium containing 10% fetal bovine serum (Gibco, USA) and 1% antibiotic (Beijing Solarbio Science & Technology, China). The cells were cultured in a Thermo Forma 3111 incubator (Thermo Fisher Scientific, USA) at 37°C with 5% CO_2_, and the medium was replaced every two days.

Immunocytochemical staining for vimentin was performed in the isolated cells to confirm their ecESC phenotype. EcESCs cultured on slides were fixed with 95% alcohol and incubated with 1:50 diluted primary mouse anti-human vimentin antibody (Boster, China) for 3 h at room temperature. After being washed with PBS 3 times, the slides were incubated with horseradish peroxidase-labeled goat anti-mouse secondary antibody (Boster) for 30 min at room temperature. The slides were stained with DAB and counterstained with hematoxylin. Finally, the slides were digitally imaged.

### Generation and transduction of lentiviruses

In our previous study (21), three shRNA interference sequences targeting lncRNA H19 were synthesized and inserted into the AgeI-EcoRI site of the pLKO.1-Puro vector. EcESCs were infected with lentiviruses containing H19 shRNA (shH19-1, shH19-2, or shH19-3) or shRNA negative control (shNC), and real-time polymerase chain reaction (RT-PCR) and western blot assays showed that the expression of shH19 in ecESCs led to decreased lncRNA H19 expression, with shH19-1 and shH19-2 showing the highest efficiency.

In this study, shH19-1 (Table 2) was selected, and pLKO.1-Puro-lncRNA-H19 shRNA or pLVX-Puro-FKBP3 was co-transfected into 293T cells with the viral packaging plasmids psPAX2 and pMD2.G (Addgene, USA) using Lipofectamine 2000 (Invitrogen, USA). After incubation for 48 h, viral particles were collected by ultracentrifugation. LV-H19-shRNA was used to infect ecESCs to knock down the expression of lncRNA H19, and the transduction efficiency of LV-H19-shRNA was evaluated using an RT-PCR assay.

**Table 2:** lncRNA H19 interference sequences.

Name	Sequence (5′ to 3′)
H19-shRNA(2357-2375)	CCAAGTAGGGACAACCCTT
H19-shRNA-Forward	CCGGTCCAAGTAGGGACAACCCTTCTCGAGAAGGGTTGTCCCTACTTGGTTTTTG
H19-shRNA-Reverse	AATTCAAAAACCAAGTAGGGACAACCCTTCTCGAGAAGGGTTGTCCCTACTTGGA

H19-shRNA: lncRNA H19 short hairpin RNA.

### Animals

Studies were conducted on 40 adult female nude mice (6-8 weeks old, 18-22 g) (Animal Experiment Center of Jiangsu University, Jiangsu University, China). Mice were raised in a specific pathogen-free (SPF) laminar flow cabinet with regulated 12-h light/dark cycles, at 23-25°C. Mice had free access to food and water and underwent a 2-week period of acclimation to the environment before the experiment.

### Mouse model of endometriosis

A nude mouse model of endometriosis was established according to a published paper (23). When infected ecESCs in good condition grew to 80-90% confluence, the cell concentration was adjusted to 1×10^8^ cells/mL after digestion and resuspension. The 40 nude mice were randomly divided into two groups, and the 20 nude mice in the experimental group were implanted with LV-H19-shRNA-infected ecESCs. The 20 nude mice in the control group were implanted with LV-NC-shRNA-infected ecESCs. On day 0, 0.2 mL of cell suspension was injected subcutaneously into the left subaxillary regions of the 40 nude mice.

After implantation, we monitored the condition of all the nude mice, the responses to external stimulation, the infection at the implantation site, and the growth of subcutaneous masses daily. The subcutaneous mass size (in mm^3^) was determined by caliper measurements performed every week and calculated by using the following formula: volume=length×width^2^×0.5. Five nude mice were sacrificed by cervical dislocation every week for 4 continuous weeks (on days 7, 14, 21, 28), and the subcutaneous masses were removed, weighed by electronic scale, and imaged. lncRNA-H19 levels were determined using RT-PCR.

All procedures were performed under isoflurane anesthesia. The tail of each mouse was marked using nontoxic permanent markers, and researchers were blinded to the treatment groups. All mice were euthanized with an anesthetic overdose at the end of the experiment.

### RT-PCR detection of lncRNA H19

RT-PCR was performed to determine lncRNA H19 expression. TRIzol reagent (Invitrogen) was used to isolate total RNA from ecESCs or endometriotic implants. One microgram of RNA was reverse transcribed into cDNA with a reverse transcription kit (Fermentas, USA). Quantitative RT-PCR analysis was carried out with a SYBR Green PCR kit (Thermo Fisher Scientific) on the ABI-7300 Real-time PCR system (Applied Biosystems, USA) under the following conditions: 95°C for 10 min; 40 cycles of 95°C for 15 s and 60°C for 45 s; 95°C for 15 s; 60°C for 1 min; 95°C for 15 s; and 60°C for 15 s. The sequences of all primers are shown in Table 3. GAPDH was used as an internal reference for the normalization of lncRNA H19 expression data. The data were analyzed by the 2^-ΔΔCt^ relative expression method.

**Table 3:** Primer sequences used in real-time polymerase chain reaction assays.

Name	Sequence (5′ to 3′)
lncRNA-H19	
Forward	GCGGGTCTGTTTCTTTACTTCC
Reverse	CTTTGATGTTGGGCTGATGAGG
GAPDH	
Forward	AATCCCATCACCATCTTC
Reverse	AGGCTGTTGTCATACTTC

### Statistical analysis

The data are reported as means±SD. GraphPad Prism 7.0 software (GraphPad Software, Inc., USA) was used to analyze the data. The statistical significance between the two groups was tested by the Student's *t*-test. A P value <0.05 was considered to be statistically significant.

## Results

### Knockdown of lncRNA H19 in ecESCs

**Figure 1:**
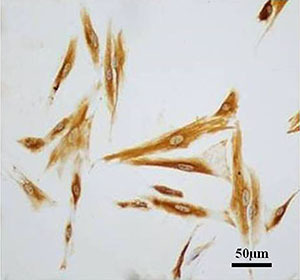
Immunocytochemical identification of endometrial stromal cells (scale bar 50 μm).

**Figure 2:**
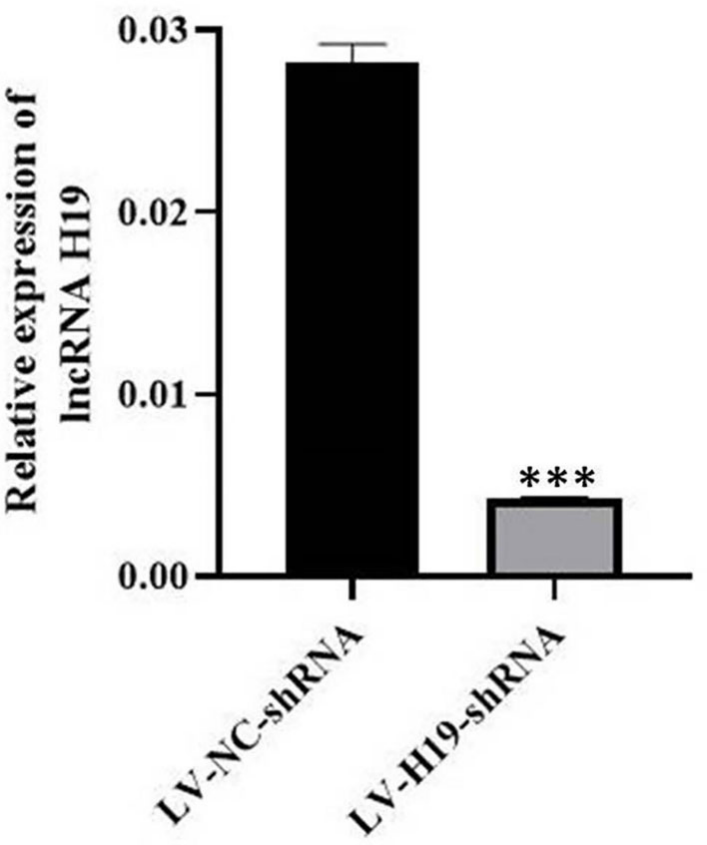
The knockdown efficiency of LV-H19-shRNA was assessed by RT-PCR in a mouse model of endometriosis. Data are reported as means±SD. ***P<0.001 compared to its negative control (LV-NC-shRNA) (Student’s *t*-test).

As shown in Figure 1, immunocytochemical results of vimentin staining confirmed that the isolated cells were ecESCs, and the purity was higher than 90%. As shown in Figure 2, infection of ecESCs from 4 patients with LV-H19-shRNA led to decreased lncRNA H19 expression. The results suggested that LV-H19-shRNA transduction knocked down lncRNA H19 in ecESCs.

### Construction of a mouse model of endometriosis

All nude mice survived the experimental period, and no significant differences in body weight were observed between the experimental group and control group. All nude mice developed histologically confirmed subcutaneous endometriotic lesions with glandular structures and stroma after 1 week of implantation. The results suggested that xenotransplantation of the human ectopic endometrium can be used to establish mouse models of endometriosis for endometriosis research.

### *In vivo* effects of lncRNA H19 in a mouse model of endometriosis

**Figure 3:**
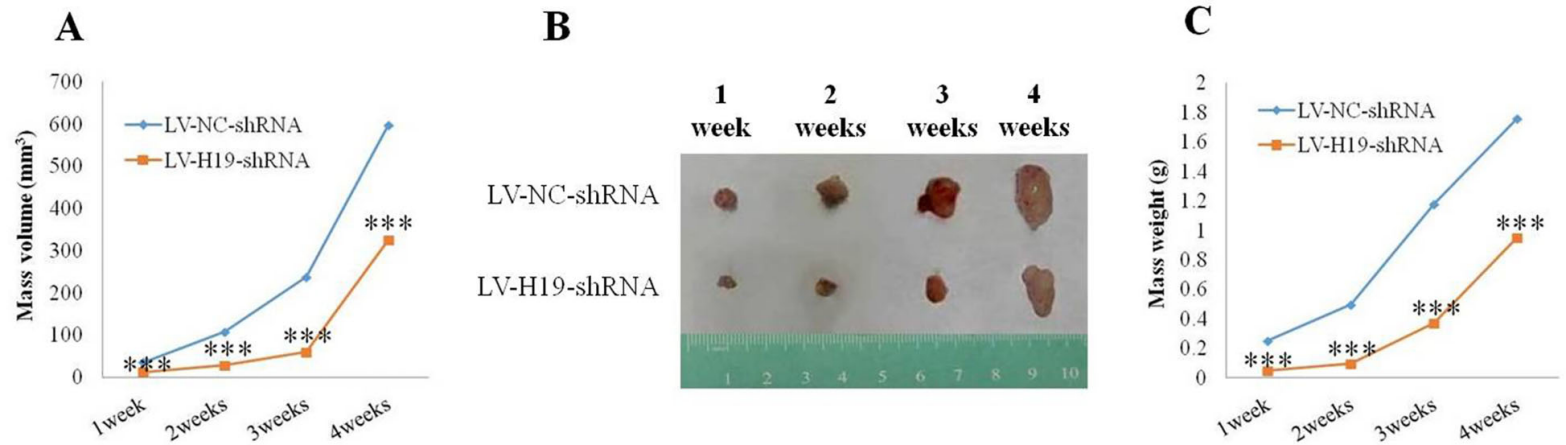
Subcutaneous lesions after 1, 2, 3, and 4 weeks of implantation of lncRNA H19 in a mouse model of endometriosis. **A**, Growth curve. **B**, Morphology (cm). **C**, Weight curve. ***P<0.001 compared to LV-NC-shRNA (negative control) (Student’s *t*-test).

**Figure 4:**
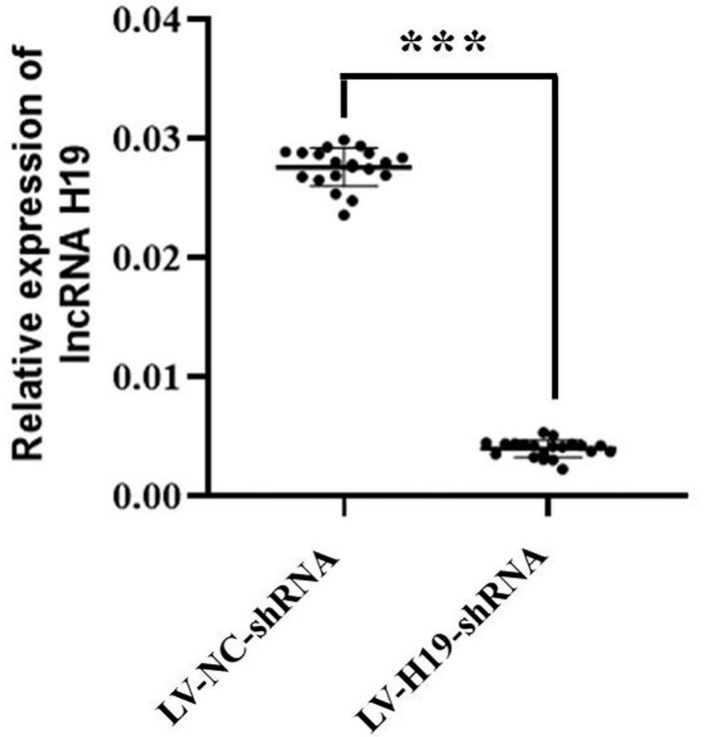
The expression of lncRNA H19 in endometriotic implants was assessed by RT-PCR. Horizontal lines indicate means±SD. ***P<0.001 compared to LV-NC-shRNA (negative control) (Student’s *t*-test).

As shown in Figure 3, the subcutaneous lesions in the LV-NC-shRNA group after 1, 2, 3, and 4 weeks of implantation were all larger than those in the LV-H19-shRNA group (P<0.001). As shown in Figure 4, the lncRNA-H19 levels in subcutaneous lesions in the LV-NC-shRNA group were significantly higher than those in the LV-H19-shRNA group (P<0.001). The results suggested that downregulation of lncRNA H19 inhibited the tumorigenicity of ecESCs in subcutaneous implants in nude mice, and knockdown of lncRNA H19 suppressed endometriosis *in vivo*.

## Discussion

lncRNA H19 is a maternally expressed imprinted gene, and is one of the earliest discovered and widely studied lncRNAs. lncRNA H19 has been shown to play important roles in many research fields. Studies of lung adenocarcinoma have reported that *in vitro*, lncRNA H19 silencing suppresses cell proliferation, sphere-forming ability, apoptosis, migration, and invasion and inhibits the epithelial-mesenchymal transition process, and *in vivo*, it also results in suppressed tumorigenicity (24). lncRNA H19 overexpression significantly promotes breast cancer cell clonogenicity, migration, and mammosphere-forming ability, while lncRNA H19 knockdown markedly inhibits tumor growth and suppresses tumorigenesis in nude mice. Mechanistically, lncRNA H19 acts as a competing endogenous RNA to sponge miRNA let-7, leading to an increase in the expression of LIN28 (25). However, there are few studies on the role of lncRNA H19 in endometriosis.

A previous study reported that decreased lncRNA H19 expression increases miRNA let-7 activity by acting as a molecular sponge and then reduces the proliferation of endometrial stromal cells via insulin-like growth factor (IGF) signaling in eutopic endometrium of women with endometriosis, which may contribute to impaired endometrial receptivity for implantation and is associated with infertility (26). Another study showed that alterations in the lncRNA H19/miR-216a-5p/ACTA2 pathway affect the invasion and migration of eutopic endometrial stromal cells and contribute to fibrous tissue formation or fibrosis in women with endometriosis. Furthermore, endometriosis is considered an estrogen-dependent disorder, and this signaling pathway has been confirmed to be regulated by estrogen (27). However, most of the existing studies focus on the eutopic endometrium, while few studies on ectopic endometrium have been carried out.

Our prior study indicated that in the ectopic endometrium of women with endometriosis, downregulation of lncRNA H19 inhibits the proliferative and invasive abilities of stromal cells through modulation of miR-124-3p and ITGB3 expression (21). Furthermore, knockdown of lncRNA H19 has been demonstrated to suppress tumorigenesis *in vivo* (24,25). Consistent with these reports, the present study also indicated that knockdown of lncRNA H19 suppressed endometriosis in nude mice. Our findings represent the first example of a function of lncRNA H19 in endometriosis *in vivo* and thus have implications for the pathogenesis and treatment of endometriosis. However, further studies of lncRNA H19-based mechanisms *in vivo* are needed in the future.

There were also some limitations that need to be addressed: 1) due to time and funding constraints, this study had a relatively small sample size; more in-depth study with a larger sample size is needed to confirm the conclusion; and 2) a nude mouse model of endometriosis was established by subcutaneous injection, and the lesions were easily observed, but the model generated by intraperitoneal injection was more similar to the endometriosis phenotype observed in humans. In the future, an intraperitoneal model could be established for further verification.

## References

[1] Yen CF, Kim MR, Lee CL (2019). Epidemiologic factors associated with endometriosis in east Asia. Gynecol Minim Invasive Ther.

[2] Liu S, Cui H, Zhang Q, Hua K (2019). Influence of early-life factors on the development of endometriosis. Eur J Contracept Reprod Health Care.

[3] Bulun SE, Yilmaz BD, Sison C, Miyazaki K, Bernardi L, Liu S (2019). Endometriosis. Endocr Rev.

[B04] Zhang NN, Sun TS, Yang Q (2019). An effective “water injection”-assisted method for excision of ovarian endometrioma by laparoscopy. Fertil Steril.

[5] Broi MGD, Ferriani RA, Navarro PA (2019). Ethiopathogenic mechanisms of endometriosis-related infertility. JBRA Assist Reprod.

[6] Shaltout MF, Elsheikhah A, Maged AM, Elsherbini MM, Zaki SS, Dahab S (2019). A randomized controlled trial of a new technique for laparoscopic management of ovarian endometriosis preventing recurrence and keeping ovarian reserve. J Ovarian Res.

[7] Fadare O, Parkash V (2019). Pathology of endometrioid and clear cell carcinoma of the ovary. Surg Pathol Clin.

[8] Kobayashi H, Yamada Y, Kawahara N, Ogawa K, Yoshimoto C (2019). Integrating modern approaches to pathogenetic concepts of malignant transformation of endometriosis. Oncol Rep.

[9] Zhou WJ, Yang HL, Shao J, Mei J, Chang KK, Zhu R (2019). Anti-inflammatory cytokines in endometriosis. Cell Mol Life Sci.

[B10] Zhang T, De Carolis C, Man GCW, Wang CC (2018). The link between immunity, autoimmunity and endometriosis: a literature update. Autoimmun Rev.

[B11] Mashayekhi P, Noruzinia M, Zeinali S, Khodaverdi S (2019). Endometriotic mesenchymal stem cells epigenetic pathogenesis: deregulation of miR-200b, miR-145, and let7b in a functional imbalanced epigenetic disease. Cell J.

[B12] Samimi M, Pourhanifeh MH, Mehdizadehkashi A, Eftekhar T, Asemi Z (2019). The role of inflammation, oxidative stress, angiogenesis, and apoptosis in the pathophysiology of endometriosis: basic science and new insights based on gene expression. J Cell Physiol.

[13] Marquardt RM, Kim TH, Shin JH, Jeong JW (2019). Progesterone and estrogen signaling in the endometrium: what goes wrong in endometriosis?. Int J Mol Sci.

[14] Mirhosseini SA, Sarfi M, Tehrani SS, Mirazakhani M, Maniati M, Amani J (2019). Modulation of cancer cell signaling by long noncoding RNAs. J Cell Biochem.

[15] Puvvula PK (2019). LncRNAs regulatory networks in cellular senescence. Int J Mol Sci.

[16] Du H, Chen Y (2019). Competing endogenous RNA networks in cervical cancer: function, mechanism and perspective. J Drug Target.

[B17] Ouyang D, Li R, Li Y, Zhu X (2019). Construction of a competitive endogenous RNA network in uterine corpus endometrial carcinoma. Med Sci Monit.

[B18] Chen Y, Bi F, An Y, Yang Q (2019). Identification of pathological grade and prognosis-associated lncRNA for ovarian cancer. J Cell Biochem.

[19] Wang WT, Sun YM, Huang W, He B, Zhao YN, Chen YQ (2016). Genome-wide long non-coding RNA analysis identified circulating LncRNAs as novel non-invasive diagnostic biomarkers for gynecological disease. Sci Rep.

[20] Liu SP, Tian X, Cui H, Zhang Q, Hua K (2019). The messenger RNA and long non-coding RNA expression profiles in ectopic and eutopic endometrium provide novel insights into endometriosis. Reprod Dev Med.

[21] Liu S, Qiu J, Tang X, Cui H, Zhang Q, Yang Q (2019). LncRNA-H19 regulates cell proliferation and invasion of ectopic endometrium by targeting ITGB3 via modulating miR-124-3p. Exp Cell Res.

[22] McGrath JC, Lilley E (2015). Implementing guidelines on reporting research using animals (ARRIVE etc.): new requirements for publication in BJP. Br J Pharmacol.

[23] Matsuzaki S, Darcha C (2014). Antifibrotic properties of epigallocatechin-3-gallate in endometriosis. Hum Reprod.

[24] Gao LM, Xu SF, Zheng Y, Wang P, Zhang L, Shi SS (2019). Long non-coding RNA H19 is responsible for the progression of lung adenocarcinoma by mediating methylation-dependent repression of CDH1 promoter. J Cell Mol Med.

[25] Peng F, Li TT, Wang KL, Xiao GQ, Wang JH, Zhao HD (2017). H19/let-7/LIN28 reciprocal negative regulatory circuit promotes breast cancer stem cell maintenance. Cell Death Dis.

[26] Ghazal S, McKinnon B, Zhou J, Mueller M, Men Y, Yang L (2015). H19 lncRNA alters stromal cell growth via IGF signaling in the endometrium of women with endometriosis. EMBO Mol Med.

[27] Xu Z, Zhang L, Yu Q, Zhang Y, Yan L, Chen ZJ (2019). The estrogen-regulated lncRNA H19/miR-216a-5p axis alters stromal cell invasion and migration via ACTA2 in endometriosis. Mol Hum Reprod.

